# Synthesis and anti-myocarditis activity in a multifunctional lanthanide microporous metal-organic framework with 1D helical chain building units

**DOI:** 10.1590/1414-431X20177050

**Published:** 2018-01-11

**Authors:** Chenglv Hong, Xinlang Zhou, Weijian Huang, Peiren Shan, Fengquan Dong

**Affiliations:** 1 Department of Cardiology, The First Affiliated Hospital of Wenzhou Medical University, Wenzhou, Zhejiang, China; 2 Department of Cardiology, Wenzhou City Hospital of Traditional Chinese Medicine and Western Medicine Combined, Wenzhou, Zhejiang, China; 3 Department of Cardiology, Shenzhen University General Hospital, Shenzhen, Guangdong, China

**Keywords:** Metal-organic framework, Anti-myocarditis, In vivo

## Abstract

A new microporous lanthanide metal-organic framework, {[Yb(BTB)(H_2_O) (DEF)_2_}_n_ (**1**, DEF=*N*,*N*-Diethylformamide), with 1D nano-sized channels has been constructed by bridging helical chain secondary building units with 1,3,5-benzenetrisbenzoic acid (H_3_BTB) ligand. Structural characterization suggests that this complex crystallizes in the hexagonal space group P6_1_22 and possesses 1D triangular channels with coordinated water molecules pointing to the channel center. In addition, anti-myocarditis properties of compound **1** were evaluated *in vivo*. The results showed that compound **1** can improve hemodynamic parameters of, and it may be a good therapeutic option for heart failure in the future.

## Introduction

Myocarditis, also known as inflammatory cardiomyopathy, is the inflammation of the heart muscle. Symptoms can include shortness of breath, chest pain, decreased ability to exercise, and an irregular heartbeat (1,2). The duration of problems can vary from hours to months. Complications may include heart failure due to dilated cardiomyopathy or cardiac arrest (3,4).

**Figure 1.**
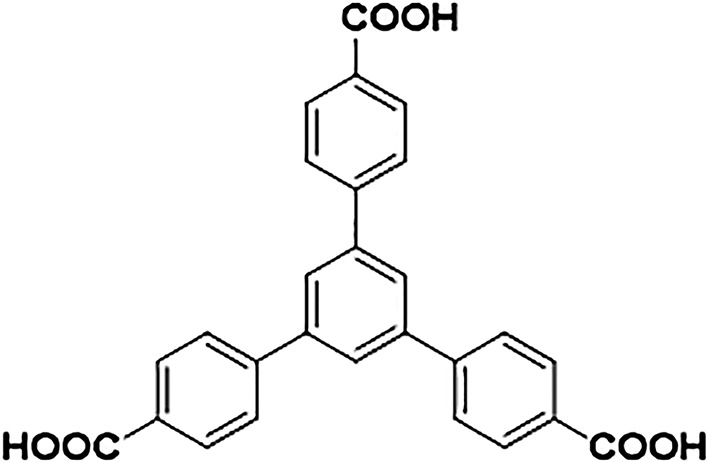
Schematic representation of the H_3_BTB ligand used in this research

Metal-organic frameworks (MOFs) that are constructed by coordination of metal centers with multiorganic connectors represent an emerging class of inorganic-organic hybrid crystalline materials (5,6). Their structural tenability, well-defined single crystal architectures, functionalized pore environment and modifiable building blocks make them useful in many potential applications including biological activity, catalysis, and luminescent sensing materials (7–9). The organic ligand plays an important role in the construction of porous MOFs because it not only guides the formation of the secondary building units, but also determines the pore shapes and pore surroundings of the obtained products (10,11). MOFs prepared with ligands of high symmetry have been well studied because of synthetic and crystallographic considerations. As the elongated ligand of H_3_BTC, 1,3,5-benzenetrisbenzoic acid (H_3_BTB, Figure 1) has been widely used in the construction of porous MOFs (12,13). However, compared with the transition metal-BTB frameworks reported, the lanthanon metal-BTB frameworks are less studied (14).

Here, we present the synthesis and the structural analysis of a highly porous Yb-organic network {Yb(BTB)(H_2_O)](DEF)_2_}_n_ (**1**, DEF=*N*,*N*-diethylformamide). This MOF is composed of novel 1D helical chain building units and BTB^3-^ ligand, which represents the first example of Ln-MOFs based on 1D helical chain building units. In addition, *in vivo* anti-myocarditis activity of compound **1** was investigated.

## Material and Methods

### Apparatus and materials

All the starting materials and reagents used in this work were obtained commercially and used without further purification. Element analyses (C, H, and N) were determined with an elemental Vairo EL III analyzer (Bruker, Germany). Powder X-ray diffraction data were collected using PANalytical X'Pert Pro powder diffractometer (Bruker) with Cu-*K*α radiation and 5° ≤2θ ≤50°. Thermogravimetric experiments were performed using a TGA/NETZSCH STA449C instrument heated from 30 to 800°C (heating rate of 10°C/min, nitrogen stream; Bruker). Single crystal X-ray diffraction was carried out by an Oxford Xcalibur E diffractometer (Bruker).

### Synthesis and characterization of {[Yb(BTB)(H_2_O)](DEF)_2_}_n_ (1)

A mixture of Yb(NO_3_)_2_·6H_2_O (0.1 mmol, 0.031 g) and H_3_BTB (35 mg, 0.062 mmol) was added to a solution of DEF (4mL) and H_2_O (1 mL). The mixture was sealed in a Pyrex tube, and heated at 140°C for 3 days. After cooling to room temperature, the colorless polyhedral-shaped crystals formed were filtered, washed with DEF, and then dried in air. Analytical data for compound **1** (C_37_H_39_N_2_O_9_Yb): C, 53.23; H, 4.44; N, 3.29%. Calculated: C, 53.62; H, 4.74; N, 3.38%.

### Crystal structure determination

Suitable single crystal of compound **1** was carefully selected under optical microscope and glued on thin glass fibers. The intensity data of **1** was collected on an Oxford Xcalibur E diffractometer. The empirical absorption corrections were applied to the data using the SADABS system. This structure was solved by direct method and refined by full-matrix least-squares method on *F* ^2^ using the SHELXS-97 program (15). All non-hydrogen atoms of **1** were refined anistropically, and all the hydrogen atoms attached to carbon atoms were fixed at their ideal positions. Pertinent crystal data and structural refinement results for compound **1** are summarized in Table 1.

**Table 1.** Crystal data and structure refinements for compound **1.**

Formula weight	624.43
Temperature/K	293 (2)
Crystal system	hexagonal
Space group	P6_1_22
a/Å	18.0081 (15)
b/Å	18.0081 (15)
c/Å	21.8141 (13)
α/°	90
β/°	90
γ/°	120
Volume/Å^3^	6126.4 (11)
Z	6
ρ_calc_g/cm^3^	1.016
μ/mm^−1^	2.316
Radiation	MoKα (λ=0.71073)
2Θ range for data collection/°	6.422 to 52.726
Reflections collected	16173
Independent reflections	4186 [R_int_=0.0485, R_sigma_=0.0462]
Data/restraints/parameters	4186/111/162
Goodness-of-fit on F^2^	1.018
Final R indexes [I>=2σ (I)]	R_1_=0.0316, ωR_2_=0.0684
Final R indexes [all data]	R_1_=0.0440, ωR_2_=0.0734
Largest diff. peak/hole / e Å^−3^	0.42/-0.78
Flack parameter	-0.026(10)
CCDC	1573543

CCDC: Cambridge Crystallographic Data Centre.

### *In vivo* anti-myocarditis activity

C57BL6/j mice were involved in our experiment. A total of 48 eight-week-old male mice were divided into four groups: control+PBS (G1, n=12), control+**1** (G2, n=12), CVB3+PBS (G3, n=12), CVB3+**1** (G4, n=12). G3 and G4 were infected by intraperitoneal (*ip*) injection of 1×10^5^ plaque forming units (*pfu*) Coxsackie virus B3 (CVB3) per mouse, while G1 and G2 received *ip* injection of phosphate-buffered saline (PBS) on the same day. Compound **1** was orally applied at 50 mg/kg on the next day of infection (G2 and G4), while G1 and G3 were orally administrated the same dose of PBS. Animals were housed with a normal diet, 12 h light/dark cycle, 30-70% humidity, and 20-25°C. All mice were sacrificed on day 7 post-CVB3 infection. We used a conductance catheter (DDS-307, Chang-Ai, China) to collect hemodynamic data (pressure and volume) before sacrificing the animals.

Statistical analysis was performed using Prism 6 (Bruker). One-way analysis of variance (ANOVA) was used for statistical analysis of the data with correction for multiple comparisons via the Tukey's range test. Data are reported as means±SD. Differences were regarded to be significant if the two-sided P-value was lower than 0.05.

## Results and Discussion

### Molecular structure

**Figure 2.**
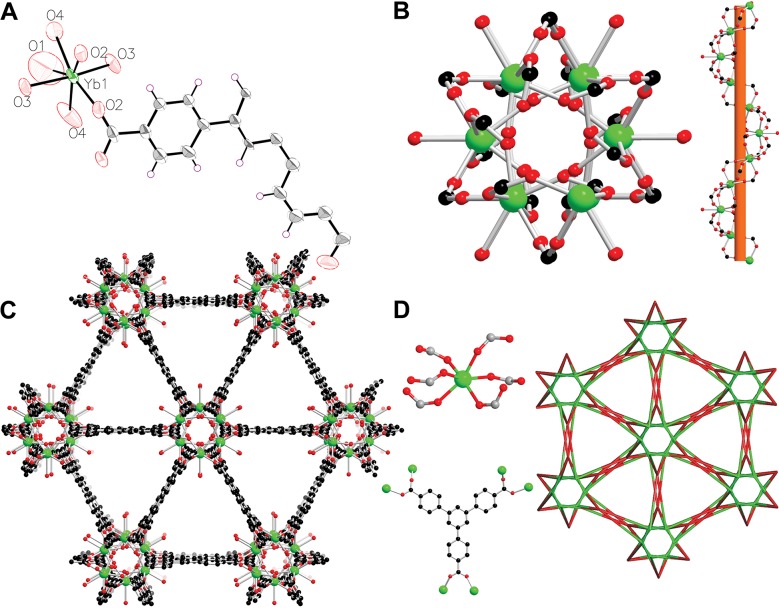
*A*, view of the asymmetric unit in compound **1** with 30% thermal ellipsoid level; *B*, view of 1D helical chain building units in **1**; *C*, view of the 1D triangular channels in **1**; *D*, (6,6)-connected topology for **1**.

The solvothermal reaction of Yb(NO_3_)_3_·6H_2_O and H_3_BTB in a mixed solvent of DEF and H_2_O provided complex **1** as colorless crystals. Single-crystal X-ray diffraction reveals that **1** crystallizes in a highly symmetric and chiral hexagonal space group P6_1_22 and the 3-D coordination network is constructed through the connection of infinite 1-D helical chain building units and the BTB^3-^ ligands. The asymmetric coordination unit consists of one Yb ion situated on a symmetry site with one half occupancy, half BTB^3-^ ligand and one coordinated water molecule. As shown in Figure 2A, the Yb(III) ion is seven-coordinated by six carboxylic acid O atoms from six different BTB^3-^ ligands and one coordinated water molecule, resulting in a pentagonal bipyramid geometry. The Yb-O bond distances are in the range of 2.212 (3) to 2.609 (5) Å. Each Yb atom is connected with the neighboring ones though three carboxylic groups. Such a connection mode leads to the formation of a 1D right-handed chain along a 6_1_ axis, which represent a rare case of Ln-based helical chain building units according to the Cambridge Crystallographic Data Centre database (Figure 2B). The pitch of the helical chain is 21.832 (3) Å. Furthermore, such 1D helical chain building units are further linked by BTB ligand through its three carboxylate groups to afford a 3D non-interpenetrating framework with 1D triangular channels with coordinated water molecule pointing to the channel center (Figure 2C). Based on the crystallographic data and considering the van der Waals radii of atoms, the pore size for the triangular channel is 5.4 Å. To understand the network of **1** more clearly, we use the software TOPOS to simplify its framework. Each Yb(III) ion is connected with six O atoms from six different BTB^-^ ligands and each BTB^-^ ligand binds with six different Yb(III) ions. Thus, both of the Yb(III) ions and the BTB^-^ ligand could be viewed as 6-connected nodes. In this case, the whole framework of **1** can be simplified to a 2-nodal (6,6)-connected network with the point symbol of (4^10^.6^5^)(4^7^.6^8^), which has not been observed in MOF chemistry (Figure 2D). The effective free volume of **1** without guest water molecules is 58.3% of the crystal volume (3569 Å^3^ of the 6126 Å^3^ unit cell volume), calculated with PLATON software.

### Powder X-ray diffraction analysis (PXRD) and thermal analysis

**Figure 3.**
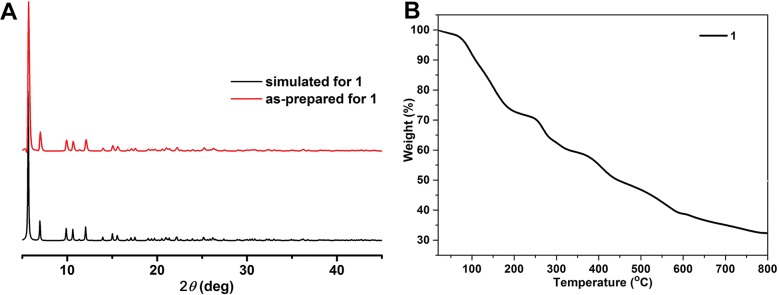
*A*, powder X-ray diffraction analysis patterns for compound **1**. *B*, TGA curve for **1**.

PXRD experiment was carried out to verify the phase purity of the as-synthesized samples. As shown in Figure 3A, the diffraction peak of compound **1** is in good agreement with that of the simulated one based on the single crystal diffraction data, indicating the pure phase of the obtained samples. From the thermogravimetric curve of compound **1**, we found that the first weight loss of 27.1% occurs from 25 to 210°C, which corresponds to the release of one coordinated water molecule and two lattice DEF molecules (Calcd: 26.5%). Then, the dissolved sample was stable up to 230°C, after which the framework began to collapse (Figure 3B).

### *In vivo* anti-myocarditis activity

To evaluate the impact of compound **1** in CVB3-induced myocarditis, body weight, heart rate (HR), maximum left ventricle pressure (Pmax), maximum left ventricle pressure rise rate (dP/dtmax), and ejection fraction (EF) were analyzed in the present study. There was a significant difference in body weight between G3 and G1. Furthermore, there was a significant difference between G4 and G3 (Table 2). In comparison to G1 and G2, there was a sharp decrease in G3 animals in HR, Pmax, dP/dtmax and EF. Oppositely, G4 had a downward trend that compared to G1 and G2, but there was no significant difference between them (Table 3).

**Table 2.** Body weight of mice at day 0 and day 7.

	Control+PBS	Control+**1**	CVB3+PBS	CVB3+**1**
Day 0 BW (g)	24.83±0.66	24.88±0.69	24.88±0.64	24.79±0.59
Day 7 BW (g)	26.65±0.49	26.37±0.74	20.41±0.73*^#^	22.41±1.15*^#+^

Data are reported as means±SD. BW: body weight. CVB3: Coxsackie virus B3; PBS: phosphate buffered saline. *P<0.01 *vs* control+PBS, ^#^P<0.01 *vs* control+**1**, ^+^P<0.01 *vs* CVB3+PBS (one-way ANOVA).

**Table 3.** Hemodynamic data of mice.

	Control+PBS	Control+**1**	CVB3+PBS	CVB3+**1**
HR (bpm)	531.48±40.32	529.98±39.46	386.77±124.59*^#^	475.79±79.13^+^
Pmax (mmHg)	113.96±21.64	109.66±12.91	90.25±22.37*^#^	105.34±11.43
dP/dtmax (mmHg/s)	9742.27±1766.32	9445.64±1920.23	6108.48±2592.93*^#^	8000.47±1378.99
EF (%)	78.60±2.65	77.76±5.20	69.66±7.74*^#^	73.64±4.59

Data are reported as means±SD. HR: heart rate; Pmax: maximum left ventricle pressure; dP/dtmax: maximum rate of rise of left ventricle pressure; EF: ejection fraction; CVB3: coxsackie virus B3; PBS: phosphate buffered saline. *P<0.05 *vs* control+PBS, ^#^P<0.05 *vs* control+compound **1**, ^+^P<0.01 *vs* CVB3+PBS (one-way ANOVA).

As known, human myocarditis can result in chest discomfort, palpitation, shortness of breath, dizziness, decreased activity, and poor appetite. CVB3-mice are a good myocarditis model that we can easily see reduced activity, and get body weight data through weighing; the decreased appetite indicates that the myocarditis model works. From our *in vivo* experiment, we found that the CVB3 group significantly lost body weight, but it seemed to reverse after application of compound **1**. The CVB3 group had a significant decrease of HR, Pmax, dP/dtmax, and EF, which are essential factors of heart failure, especially systolic heart failure. In our investigation, compound **1** was effective in hemodynamics, indicating it could be a candidate for anti-myocarditis therapy.

In conclusion, we demonstrated the successful construction of a novel Yb-based MOF with 1D helical chain building units built up from 1,3,5-H_3_BTB ligand. Structural characterization suggests that this complex crystallizes in the hexagonal space group P6_1_22 and possesses 1D triangular channels with coordinated water molecules pointing to the channel center. In addition, the results showed that compound **1** can improve hemodynamic parameters, and may be a good therapeutic compound for heart failure in the future.
